# Immigrant and refugee health.

**DOI:** 10.3201/eid0403.980323

**Published:** 1998

**Authors:** S Cookson, R Waldman, B Gushulak, D MacPherson, F Burkle, C Paquet, E Kliewer, P Walker

**Affiliations:** Centers for Disease Control and Prevention, Atlanta, Georgia, USA.


### Immigrant and Refugee Health

**Susan Cookson,* Ronald Waldman,* Brian Gushulak,† Douglas MacPherson,‡ Frederick Burkle, Jr.,§ Christophe Paquet,¶ Erich Kliewer,# and Patricia Walker****

*Centers for Disease Control and Prevention, Atlanta, Georgia, USA; †The International Organization for Migration, Geneva, Switzerland; ‡St. Joseph's Hospital, Hamilton, Ontario, Canada; §University of Hawaii, Honolulu, Hawaii, USA; ¶Epicentre, Paris, France; #Manitoba Cancer Treatment and Research Foundation, Winnipeg, Manitoba, Canada; and **Regions Hospital, St. Paul, Minnesota, USA

Each year, more than 15 million people seek political asylum or become refugees in various parts of the world. Most of these displaced persons are from developing countries where infectious diseases (e.g., tuberculosis, hepatitis, malaria, various parasitic and emerging diseases) are prevalent. These persons migrate mainly to the United States, Australia, and Canada, nations that receive inflows of migrants proportional to their mainstream population.

Because of the speed and efficiency of modern transportation systems, health interventions applicable to all persons who cross international borders are difficult to introduce and monitor. Identifying and addressing individual and public health risks necessitate international and quarantine health legislation, health policy and social economic evaluation, risk-benefit and utility analysis, and risk-predictive modeling. Ultimately, improving the health of migrants is at the heart of reducing the public health risk to the international community from infectious disease spread by travel.

Medical intelligence systems that can survey, detect, and confirm the emergence of new infectious diseases are still in their infancy. The global ability to generate numerators (cases of existing and emerging infectious disease) has been limited to the relatively few diseases listed in the old World Health Organization (WHO) International Health Regulations (yellow fever, plague, cholera, smallpox); further limitations stem from poor detection systems and incomplete reports (with the exception of smallpox). The dynamic problem of defining numerators and denominators (displaced persons at risk) is compounded by the need for improved diagnostics, heightened recognition, and effective medical interventions for the causative agents of diseases that affect these vulnerable populations.

Migrant populations have been displaced by disasters (natural, technologic, and human), which test the public health resources of a nation and expose weaknesses. Public health workers increasingly appreciate the fragile interaction between individual host, environment, and infectious and noninfectious agents capable of producing disease. The consequences of these relationships, including the real and potential vulnerability of populations, are becoming increasingly important indicators of national security.

Cholera, a disease that affects migrant populations, was examined. In Malawi, 11 outbreaks were documented in Mozambican refugees between 1987 and 1991, with attack rates of 0.6% to 9.3%. In 1994, an estimated 60,000 cases of cholera and 10,000 deaths occurred during a 1-month massive epidemic among Rwandan refugees (population 800,000) in Goma, Democratic Republic of Congo. Epidemic preparedness during the 1996 return of the epidemic proved the cornerstone of cholera control in these refugees. Properly implemented, active case-finding and rehydration therapy in specialized treatment centers can keep the case-fatality ratio below 1%.

In Australia, use of hospital and medical services by immigrants and refugees was examined. Foreign-born persons had lower hospitalization rates than native Australians, although some immigrant groups had higher rates for some diagnoses. Hospital data may help define trends in immigrant disease profiles; the data, however, do not indicate whether the generally lower hospitalization rates among immigrants were due to better health status or to barriers in accessing the medical system. In the

United States, Minnesota has been a leader in refugee resettlement since 1979; one center for international health has established a unique multidisciplinary primary and specialty care program for refugees and immigrants. Hmong, Cambodian, Vietnamese, Russian, Ukrainian, African, and Latin American refugees and immigrants have been seen; diseases such as hepatitis B, tuberculosis, and parasitic diseases, as well as mental health problems, have been diagnosed; and prevention strategies or therapies have been implemented.

Successful integration of migrant populations into their new communities' health-care systems is critical to the prevention and control of new and reemerging infectious diseases.

